# Genome Sequence of *Bacillus cereus* Phage vB_BceS-MY192

**DOI:** 10.1128/genomeA.01557-15

**Published:** 2016-04-21

**Authors:** Yong Yang, Li Zhan, Jiancai Chen, Yunyi Zhang, Yi Sun, Zhangnv Yang, Liping Jiang, Hanping Zhu, Yanjun Zhang, Yiyu Lu, Lingling Mei

**Affiliations:** Department of Microbiology, Zhejiang Provincial Center for Disease Control and Prevention, Hangzhou, Zhejiang, China

## Abstract

*Bacillus cereus* is an opportunistic foodborne pathogen. The phage vB_BceS-MY192 was isolated from *B. cereus* 192 in a cooked rice sample. The temperate phage belongs to the *Siphoviridae* family, *Caudovirales* order. Here we announce the phage genome sequence and its annotation, which may expand the understanding of *B. cereus* siphophages.

## GENOME ANNOUNCEMENT

*Bacillus cereus* is a spore-forming soil bacterium that propagates in many kinds of foodstuffs and products. It causes food poisoning with symptoms of vomiting and diarrhea (1). Since bacteriophages can be used to control bacterial pathogens and type different strains and are useful in the fermentation industry, many phages have been isolated and intensively investigated. However, the isolation and study of phages in *B. cereus* are still far from complete (2–4). One phage has been detected and isolated in its natural host, the *B. cereus* 192 strain from a cooked rice sample. The phage killed *B. cereus* 192 and because the phage was in its lytic stage it made large and clear plaques in the bacterial colony. This phage was morphologically identified by TEM and found to belong to the *Siphoviridae* family, *Caudovirales* order (5, 6). The siphophage was named vB_BceS-MY192 according to the nomenclature for bacteriophages proposed by Kropinski et al. (7).

The genome of host strain *B. cereus* 192 with the prophage vB_BceS-MY192 was sequenced using an Illumina genome sequencer. The assembly of quality filtered reads was performed using MaSuRCA V. 2.3.0, and the predictions of open reading frames (ORFs) and their confirmation were conducted using the ORF Finder (http://www.ncbi.nlm.nih.gov/gorf/gorf.html) and GeneMark.hmm-P (version 2.5), respectively (8, 9). Conserved protein domain analysis of predicted ORFs was carried out using BLASTP (10, 11). A total of 707 Mb of high-quality paired-end sequences from DNA libraries with a size of 500 bp were generated. This represented approximately 101× coverage of the bacterial genome, which was estimated to be ~7 Mb. Also, the coverage of the phage genome was estimated to be 101×. The genome sequence of vB_BceS-MY192, separated from the host strain whole-genome sequence, showed a 44,696-bp length, 64 coding sequences (CDS), 171 ORFs, and a distinct high GC content of 53.80%. Since no permuted, direct repeat, cos, or protein-primed ends were found in the genome sequence, the sequence is considered partial and must be completed in the future. BLASTN of the genome against the Genbank database revealed that the “HNH endoneuclease” and “phage tail family protein” were highly homologous to those of *B. cereus* sensu lato prophages. It also showed that the sequence of phage phIS3501 with the highest maximum score among the *Bacillus* phages was 87% identical, with only 12% query coverage of the genome sequence, which means the vB_BceS-MY192 was a novel phage targeting *B. cereus*. It was also discovered that the lysogenic vB_BceS-MY192 contributed to the virulence of *B. cereus* 192 (data not shown).

This phage genome encodes structural and packaging proteins, such as phage capsid protein, tail protein, phage head-tail adapter protein, phage tail length tape measure protein, and integrase (12, 13). There are Clp protease proteolytic subunit, XRE, and ArpU family transcription regulators, sigma 70 factor, and holin in this genome, suggesting that the siphophage might complete more functions other than synthesizing its own structural proteins and DNA (14, 15). Furthermore, this genome encodes many unidentified hypothetical proteins (63%), suggesting there are many questions to be explored in this phage.

### Nucleotide sequence accession numbers.

This whole-genome shotgun project has been deposited in DDBJ/ENA/GenBank under the accession no. KT725776. The version described in this paper is the first version, KT725776.1.
